# Excitatory Local Interneurons Enhance Tuning of Sensory Information

**DOI:** 10.1371/journal.pcbi.1002563

**Published:** 2012-07-12

**Authors:** Collins Assisi, Mark Stopfer, Maxim Bazhenov

**Affiliations:** 1 Department of Cell Biology and Neuroscience, University of California, Riverside, California, United States of America; 2 US National Institutes of Health, National Institute of Child Health and Human Development, Bethesda, Maryland, United States of America; Indiana University, United States of America

## Abstract

Neurons in the insect antennal lobe represent odors as spatiotemporal patterns of activity that unfold over multiple time scales. As these patterns unspool they decrease the overlap between odor representations and thereby increase the ability of the olfactory system to discriminate odors. Using a realistic model of the insect antennal lobe we examined two competing components of this process –lateral excitation from local excitatory interneurons, and slow inhibition from local inhibitory interneurons. We found that lateral excitation amplified differences between representations of similar odors by recruiting projection neurons that did not receive direct input from olfactory receptors. However, this increased sensitivity also amplified noisy variations in input and compromised the ability of the system to respond reliably to multiple presentations of the same odor. Slow inhibition curtailed the spread of projection neuron activity and increased response reliability. These competing influences must be finely balanced in order to decorrelate odor representations.

## Author Summary

The antennal lobe of insects and the olfactory bulb of vertebrates represent the first centers of the olfactory system where information about odor properties can be reorganized and optimized for further processing. Complex excitatory and inhibitory synaptic interactions within the antennal lobe and the olfactory bulb alter the responses of the principal neurons throughout the duration of the odor stimulation. These dynamic changes progressively increase the difference between firing patterns evoked by structurally similar odors, potentially helping the animal distinguish one odor from another. However, this process, called odor decorrelation, appears to oppose another important goal of olfactory processing, to minimize the inevitable noisy variations in representations of the same odor encountered under different environmental conditions; such variations could potentially lead to misclassification. It remains an interesting mystery how olfactory circuitry can solve these two seemingly contradictory goals as they process olfactory stimuli: first, separating different but chemically similar odors (sensitivity, capacity); and second, identifying representations of the same odor in a noisy environment (reliability). Our results suggest a balance between inhibitory and excitatory connections mediated by local antennal lobe interneurons enhances the decorrelation of similar odors while keeping the representation robust in the presence of noise.

## Introduction

The olfactory system must accomplish two seemingly conflicting goals —generate distinct representations of different odors, yet maintain stable representations of a repeated odor despite variability introduced by noise. These conflicting ends, separability and reliability, are met as information about odors traverses multiple levels of the olfactory system.

Odor detection begins when odorant molecules bind to olfactory receptor neurons (ORNs) and initiate cellular mechanisms leading to the opening of ion channels, the depolarization of the receptor neuron cell membrane, and the generation of action potentials [1]. Because each receptor type responds better to some odorants than others, the representation of an odor can be described as a spectrum of activation patterns across the receptor population [2], [3], [4]. Similar odors are presumably represented by similarly distributed patterns of activation [5]. Information about most odors, to a first approximation, is encoded in ORNs in a combinatorial manner [6]. In insects, these patterns of receptor activation are then conveyed to an olfactory structure called the antennal lobe (AL), which contains far fewer neurons than there are ORNs (in the locust, for example, ∼90,000 ORNs converge onto just 830 projection neurons (PNs) and 300 local inhibitory interneurons (LNs) [7]).

Antennal lobe neurons respond to odor-elicited input with a rich variety of spatiotemporal patterns [8], [9], [10], [11]. Many investigators, beginning with Adrian [12], have suggested the temporal pattern of spiking in these second order neurons encodes information about odor quality. These spatiotemporal patterns of activation unfold along multiple spatial and temporal scales, transiently and successively recruiting different groups of neurons, contributing to the progressive decrease in the overlap between odor representations in the AL [11], [13], [14].

What network interactions shape spatio temporal patterning in the AL to accomplish essentially opposed information processing goals: that representations of different odors may be rapidly distinguished; yet the same odor presented under changing environmental circumstances is reliably identified? To address this question we examined the contributions of two factors in a realistic model of the locust AL [15], [16]: 1) lateral local excitation between PNs mediated by putative local excitatory interneurons [17], [18], [19]; and 2) slow inhibition from LNs to PNs [15], [20]. We propose that these two complementary excitatory and inhibitory influences must be optimized to achieve both reliable and separable odor representations.

## Results

### Complementary effects of lateral excitation and slow inhibition on Antennal Lobe dynamics

**Figure 1:**
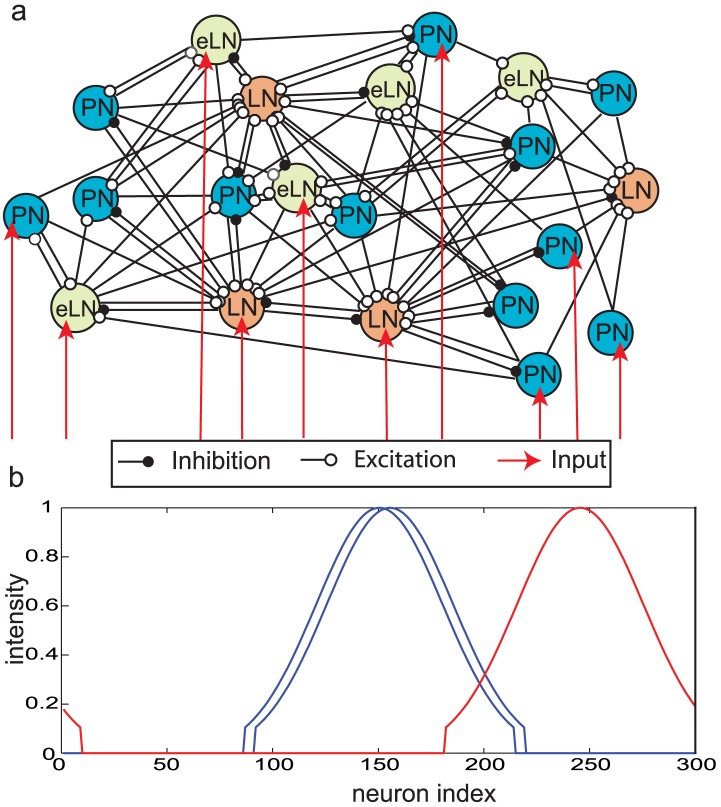
**a.** Schematic diagram of the antennal lobe network consisting of projection neurons (PNs), inhibitory local interneurons (LNs) and excitatory local interneurons (eLNs). (See text for connection probabilities between different neuron types). A random sampling of neurons receives external input (red arrows). **b.** Input to the neurons. Each PN, LN, and eLN receives external input with amplitude chosen from a truncated Gaussian distribution. The similarity between odors can be varied by changing the overlap in the input profile to individual neurons. The Gaussian intensity profile of similar odors (compare the blue lines) show a large overlap whereas dissimilar odors (compare blue and red lines) show very little overlap. (Note that the red profile “wraps around” from right to left.).

In the insect olfactory system input from ORNs converges into PNs and LNs of the AL. With a model of the AL network we sought to test the complementary effects of fast lateral excitation and slow inhibition, both of which have been observed *in vivo*. The model network was based on locust anatomy and consisted of reciprocally connected PNs and LNs. Lateral excitation was implemented by a class of excitatory interneurons (eLNs) that have been described in the AL of *Drosophila* [17], [18], [19]. Although experiments to directly label eLNs have not yet been performed in the locust, we infer they exist in this species from their prevalence in other insect species (fly [21], moth [22]), and from the broadly-tuned and complex responses of locust PNs consistent with widespread excitation. In the model eLNs receive input from both the PN and LNs and also provide lateral excitation to both these classes of neurons (see Figure 1a for a schematic diagram of the network architecture).

To test the network's responses to external input we simulated two classes of odor stimuli: odors represented by blue traces in Figure 1b are similar to each other (they activate overlapping sets of neurons) but are highly dissimilar to the odor represented by the red trace(see Methods). Input was provided to PNs, LNs and eLNs.

**Figure 2:**
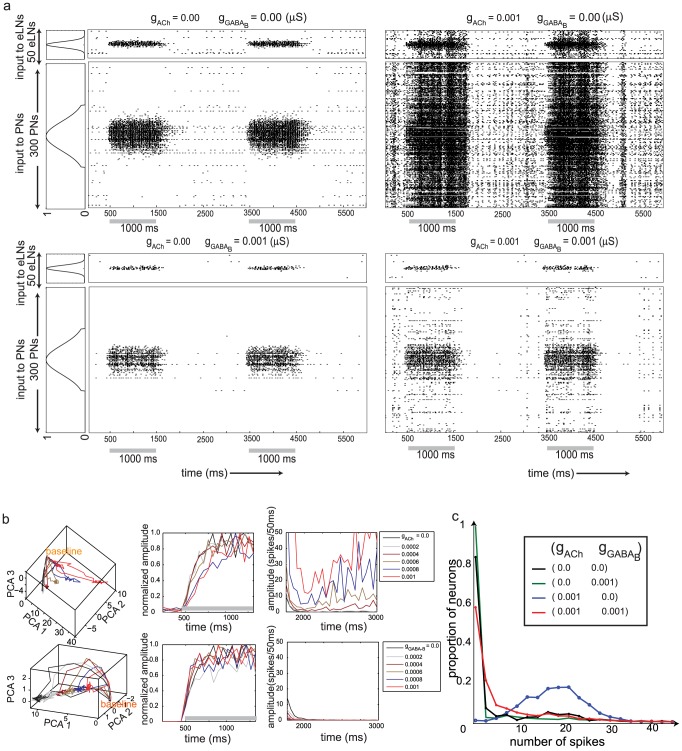
**a.** Raster plots showing the effect of lateral excitation and slow inhibition on network dynamics. In the absence of lateral excitation, the neuronal activity is largely driven by the input (top left). Large values of lateral excitation allow input to explosively and unrealistically recruit the entire network (top right). Adding slow inhibition curtails this activity (bottom right). The figure shows both the PNs (n = 300) and the eLNs (n = 50). The width of input to the eLNs is a scaled down from that of the PNs. The trace on the left of the plot shows the maximum amplitude of the input to PNs and eLNs. The onset of the input is indicated by the gray bars along the time axis. Two odor presentations, each lasting 1000 ms, are shown. **b.** Left panels. Traces show the temporal evolution of the first three principal components generated from peri–stimulus time histograms as a function of increasing lateral excitation (top panel excitation increases from blue to red, 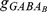 = 0.0002 is constant) and slow inhibition (bottom panel inhibition increases from blue to red, 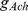 = 0.0002 is constant). Middle Panels. Normalized amplitude of the traces shown in the left panels as a function of time. Different color traces correspond to different values of lateral excitation (top panel) or slow inhibition (bottom panel). The gray bar indicates an odor presentation. Right panels. The amplitude of the traces shown in the left panels as a function of time. Here the amplitude is not normalized and the responses are shown following odor offset. **c.** Response distribution of PNs. The proportion of PNs generating a given number of spikes during odor stimulation is shown for different values of lateral excitation and slow inhibition.

We reasoned that unrestricted lateral excitation within the AL could potentially recruit neurons explosively, and we hypothesized that slow inhibition (mediated by GABA_B_ receptors) could provide both a suitable counterbalance to this, and an ability to generate broadly distributed, temporally structured responses in the PN ensemble. Indeed, our simulations showed that a balance of lateral excitation and slow inhibition prevented cascading excitation that could recruit all neurons in the network, and at the same time allowed some neurons that receive sub–threshold input directly from ORNs to become activated (Figure 2a, compare top left and bottom right panels). Note that fast lateral inhibition mediated by GABA_A_ receptors was present in all the simulations including those in which slow inhibition was removed (Figure 2a, top left). Fast inhibition is responsible for the suppression of PN responses in Figure 2a, top left, despite the lack of GABA_B_ mediated slow inhibition. To visualize these population-wide responses, we calculated the peri–stimulus time histogram (PSTH) for each PN and projected the collective dynamics of the model's three hundred PNs onto the first three principal components (Figure 2b). Before stimulus onset the trajectories wandered near the baseline (marked in Figure 2b). Upon odor stimulation the trajectory moved toward a state defined by increased population activity. The trajectory then returned to the baseline following odor termination. As the strength of lateral excitation increased, the resulting trajectories swept out increasingly wide loops (Figure 2b, top panel), indicating stronger population responses. Increasing the strength of slow inhibition had the opposite effect (Figure 2b, bottom panel), a general decrease in the activity of PNs. Further, we examined the time taken for a stimulus to push the system to its maximal distance from baseline. Because the response amplitude was determined by the number of PNs that were recruited during odor stimulation, active state properties varied with values of lateral excitation and slow inhibition. To compare the speed with which networks with different characteristics reached maximum response amplitude, we normalized the amplitude of the trajectories by the maximum amplitude and plotted the different traces as a function of time. We found that increasing lateral excitation increased the maximum value of the response amplitude (data not shown). The time taken by the system to arrive at its maximum distance (compare the traces in Figure 2b middle panel) also increased with increasing excitation. Increasing slow inhibition, on the other hand, caused a less pronounced effect in the opposite direction. We also found that the baseline shifted as a function of lateral excitation since more PNs were active even in the absence of an odor stimulus (Figure 2b right). A histogram of the responses of PNs (Figure 2c) showed that, in the absence of lateral excitation, very few neurons generated more than 10 spikes during an odor presentation (green and black traces). The green trace shows that in the absence of lateral excitation, at the highest value of slow inhibition simulated here (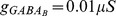), most of the PNs remained silent except for those receiving supra–threshold input. When lateral excitation was increased in the model, the response distribution shifted toward higher density spiking (blue trace). Introducing slow inhibition modulated the spread of activity caused by excitation (red trace).

**Figure 3:**
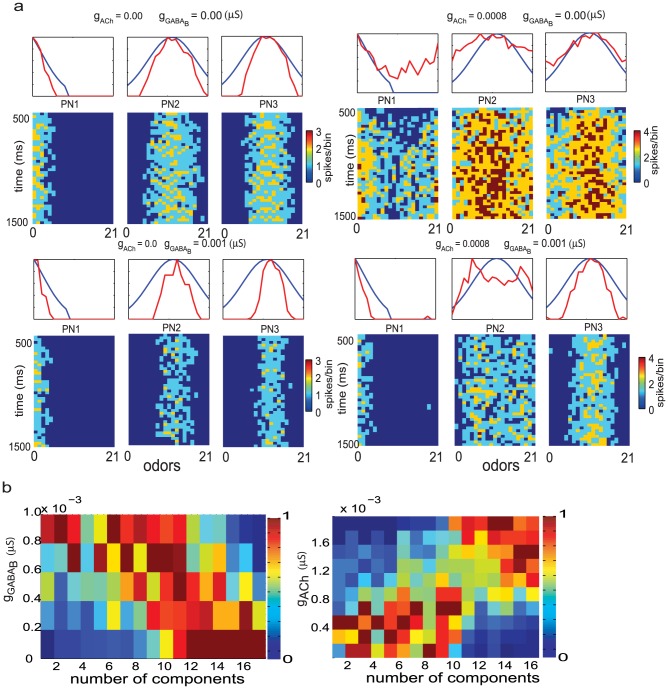
**a.** Each group of panels shows the activity of a representative set of three neurons. The image map shows the spike activity evolving over the duration of the stimulus presentation (500 to 1500 ms) in response to an array of 21 odors averaged over ten repetitions of each odor. The average normalized activity over this duration is shown by the red trace in the panel above. The blue trace shows the normalized amplitude of the input to the neuron for the set of 21 odors. The responses to different combinations of lateral excitation and slow inhibition are shown. **b.** Complexity of PN responses. The temporal spiking patterns for individual neurons over an array of 21 odors (see panel *a* for examples) were chosen and the number principal components required to explain 80% of the variance of each such pattern was calculated. For each value of lateral excitation and slow inhibition, this generated 300PNs×10trials = 3000 such numbers estimating the complexity of each PNs response to the array of odors. The normalized distribution of this number is shown as a function of lateral excitation and slow inhibition.

Next, to characterize each PN's tuning properties we simulated a broad range of odors by successively displacing the Gaussian input (Figure 1b) by five unit steps. Figure 3a shows the response of a representative set of three PNs to an array of 21 odors; the input each PN received as a function of 21 different odorants is plotted as a blue trace in the top panels. We found the responses of PNs to this array of odors (red traces) depended on the amount of lateral excitation and slow inhibition. In the absence of both types of lateral input (top left panels), responses of PNs were driven entirely by the input simulating the activity of ORNs, and each PN responded only when it received direct supra–threshold input. However, consistent with recent studies in the fly showing that PNs not receiving direct input from ORNs may be activated by indirect input from other PNs via lateral excitation [18], our model showed that, as the value of lateral excitation was increased, responses of PNs became less selective (the mean response over the duration of the odor presentation is shown by the red trace) (Figure 3a, top right panels). Beyond broadening the response to an array of odors, the addition of lateral excitation led to qualitative changes in the shape of the response curve (for example, PN1 in the top right panels started to show strong response to odors 15–21, Figure 3a). Thus, the output of PNs may interact with the input from ORNs in a nonlinear manner. Increasing slow inhibition caused opposite effects in PNs, narrowing the widths of their tuning curves (Figure 3a, bottom left panels). Including both lateral excitation and slow inhibition in the model allowed PNs to respond reliably and specifically; however, it also allowed a nonlinear remapping of the output of ORNs to the responses of PNs. This nonlinear transformation of ORN activity is evident in Figure 3a (compare top left and bottom right panels). The example PNs shown in Figure 3a were chosen to show the range of response patterns observed in PNs given different parameter values. The specific proportions of each type of response varied considerably across the range of parameter values.

Further, we analyzed the roles lateral excitation and slow inhibition play to shape the complexity of spatiotemporal responses of PNs to an odor presentation. We first determined each PN's response to a panel of 21 odors (similar to each panel in Figure 3a). To provide a measure of the complexity of the response pattern of each PN to an array of odors we then used the following procedure. For a given PN, the response to each odor of a set of 21 odors was binned (50 msec bins). The PN's response to the entire odor set was represented as a trajectory in a 21 dimensional space (each dimension corresponding to one of the odors). If the PN's response remained static over the duration of the odor presentation, then the trajectory would appear as a single point in this space. The complexity of this trajectory reflected both the diversity of the PN's responses to the set of 21 odors and the variability of these responses over time. We computed the principal components of the [21 odors ×20 time bins] array and then determined the variance explained by each 21 dimensional principal component (given by its eigen value).We then computed the number of principal components required to explain at least 80% of the variance observed in PN response patterns. This number provided a measure of the complexity of the response pattern of each PN to an array of odors. We calculated this number for each of 300 PNs and 10 presentations of each odor stimulus, with varying amounts of lateral excitation and slow inhibition. Thus, we obtained 3000 measurements to assess the complexity of the network response. Finally, we plotted the normalized distribution of these values for different amounts of lateral excitation and slow inhibition. By normalizing the histogram for each value of lateral excitation and slow inhibition, we were able to detect a trend in the peak location of each histogram despite changes in the height of the distribution. Figure 3b shows that, as the strength of lateral excitation grew, the peak of the distribution shifted to higher values, indicating an increase in the complexity in the responses of PNs. Again, slow inhibition had the opposite effect – it led to the recruitment of fewer neurons during odor stimulation, particularly in the absence of lateral excitation (see Figure 3a, bottom left). Note, however, that for larger values of slow inhibition the distribution also became broader. This suggests that while the activity of some PNs was suppressed and became less complex, the activity of other PNs remained diverse across odors and variable over time even given the greatest strength of slow inhibition.

### Decorrelation of odor representations caused by network interactions

Decorrelation of odor representations, a process that reduces the overlap between odors, occurs over the duration of the stimulus presentation. Network interactions between PNs and LNs likely play a crucial role in this process. In this study AL neurons received a stable pattern of input from the ORNs. If the AL neurons respond to this input by generating a spatially distributed but static pattern of activation, then the pattern should not decorrelate over time. Decorrelation over time is only possible if the odor representation is transformed either by network interactions or by temporally varying noise.

**Figure 4:**
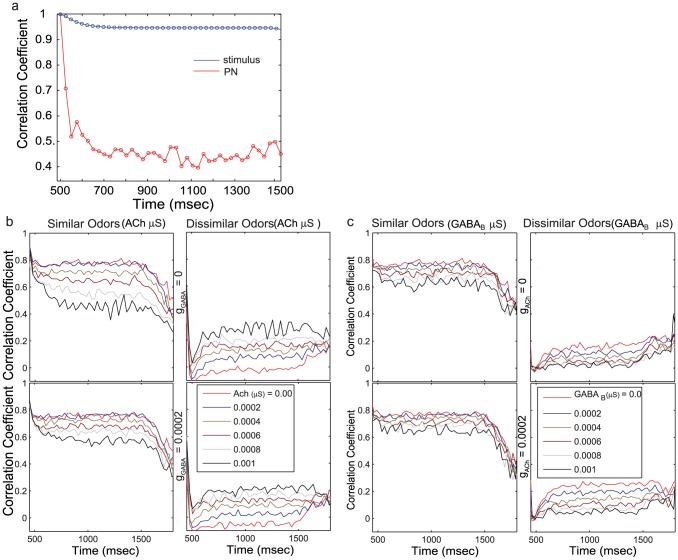
**a.** The correlation between the 300-D activity vector at the onset of the odor stimulus and the activity at subsequent 50 ms epochs decreases progressively (red trace). The blue trace shows the correlation between the input to the network at the onset of the odor and subsequent points in time. **b.** Average correlation between 300-D activity vectors over time for similar odors (left panels) and dissimilar odors (right panels) for two values of slow inhibition (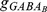 = 0 and 0.0002 µS) and a range of values of lateral excitation. Lateral excitation was required to observe measurable decorrelation. Odor stimulation was applied at 500 msec. Note that correlations start to change before t = 500 msec, because 50 msec time bins were used (see methods). Representative time interval of the correlation coefficient change during odor stimulation includes t = [500 msec, 1500 msec]. **c.** Average correlation between 300-D activity vectors over time for similar (left panels) and dissimilar (right panels) odors for two values of lateral excitation (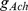 = 0 and 0.0002 µS) and a range of values of slow inhibition.

To determine the degree to which noise can play a role in transforming the odor representation, we first calculated the correlation coefficient between the onset and subsequent epochs of the input vector provided to the PNs (Figure 4a, blue line). We then compared this value with the correlation between the initial responses of 300 PNs to an odor and their responses at subsequent times (Figure 4a, red line). We found that the correlation between the onset and subsequent epochs of the odor response by PNs decreased dramatically over the first 200 ms (Figure 4a, red line) whereas the correlation between the onset and subsequent epochs of the input vector decayed to a far lesser extent. This result demonstrates that network interactions within the AL play a crucial role in reducing the similarity between responses of PNs at the odor onset and at later points in time [14], [23] and that the decorrelation is not driven entirely by noise.

Next, we sought to characterize the ability of the population of PNs to differentiate among different odors. We presented a set of 21 odors and calculated the correlation between the responses of PNs to any two odors over time. Together, the correlation coefficients for each 50 ms time window formed a 21×21 matrix. To analyze the AL mechanism responsible for this decorrelation we then calculated the change over time in the correlation coefficient averaged for all similar and, separately, for all dissimilar odors, as a function of increasing amounts of lateral excitation and slow inhibition (Figure 4b,c). We found that over the duration of the odor presentation, responses of the PN ensemble to similar odors became progressively different from one another, as evident in the decreasing correlation coefficients plotted in Figure 4b. In contrast, correlations between responses to dissimilar odors progressively increased (also compare the left and right panels in Figure 4b over the range from ∼500 to 1500 ms). Note, that in the absence of odor input (<500 ms in our simulations) very few neurons generated spikes, resulting in activity vectors with many zero elements. The correlations between these vectors therefore tended to be high (∼1.0). Overall, our findings are in a good agreement with results previously obtained *in vivo* from the zebrafish olfactory bulb [14]. The decrease in correlation can be attributed in large part to the dynamical behavior of the AL circuitry since a similar decrease is not observed in the input to PNs and LNs (Figure 4a, top panel); and noise in the input should not play a significant role in decorrelating the odor responses.

For similar odors we found that increasing the amount of lateral excitation lead to a decrease in the correlation between odor responses at a given time (Figure 4b, left panels). Increasing the strength of slow inhibition led to a decrease in the correlation between responses, but to a lesser extent than that seen when lateral excitation was increased (Figure 4c). Surprisingly, we observed the opposite in the correlation between dissimilar odors (Figure 4b, right panels). The increase in correlation over time for dissimilar odors may be attributed to the fact that the dissimilar odors were maximally decorrelated to begin with (note that the correlation coefficient at the odor onset (∼500 ms is nearly 0). Lateral excitation tended to recruit additional neurons that were not activated in the absence of excitation (compare bottom left and bottom right panels of Figure 2a). Increasing lateral excitation would increase overlap in the population of neurons recruited by a given odor, thus increasing the correlation coefficient. Indeed, as the strength of lateral excitation increased from 0.0 µS to 0.001 µS and the degree of overlap presumably increased, the correlation between dissimilar odors also increased (Figure 4b, right panel, compare lines of different color). In contrast, upon increasing the strength of slow inhibition, the overlap between the sets of neurons representing dissimilar odors decreased and led to a concomitant decrease in the correlation (Figure 4c, right panels). Recordings made in vivo from mitral cells in zebrafish olfactory bulb also showed a similar trend [14].

### Relative contributions of overall PN activity vs. complexity of the PN response pattern to odor response decorrelation

**Figure 5:**
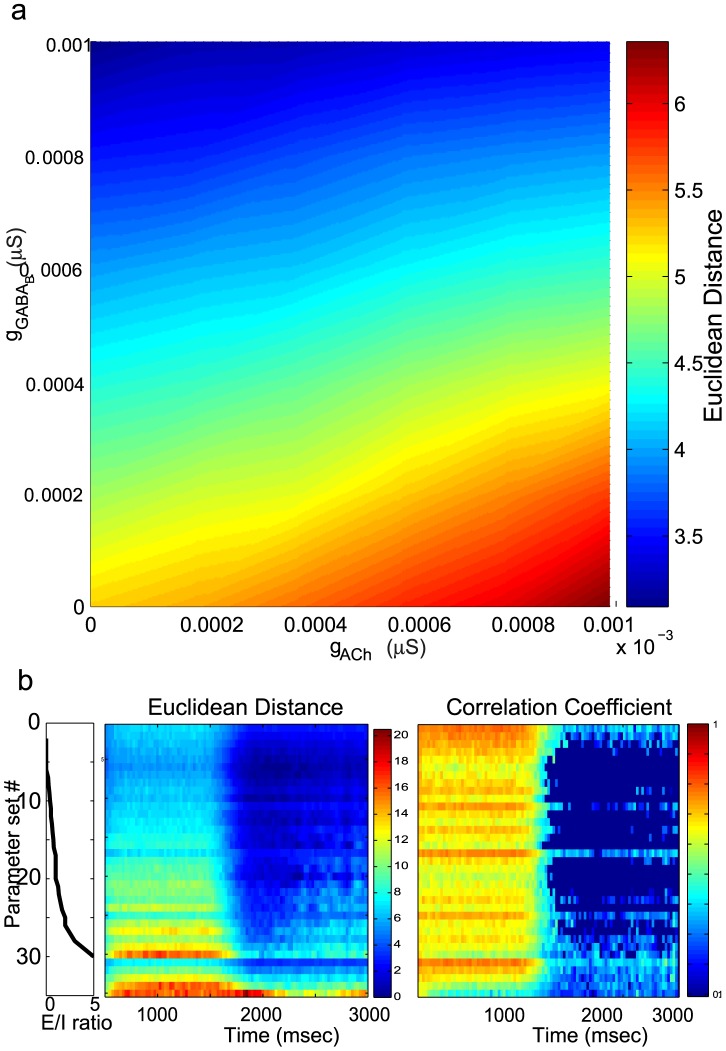
**a.** The mean Euclidean distance between the representations of similar odors shown as a function of lateral excitation and slow inhibition. **b.** Time series of the Euclidean distance and correlation coefficient between similar odors arranged in order of the excitation-to-inhibition (E/I) ratio. The leftmost panel shows the value of the E/I ratio (x-axis) for different parameter sets (y-axis). When the denominator was zero we set the value of the ratio to 5 and arranged it according to increasing strength of lateral excitation. The right panels show the change in the distance and the correlation coefficient from odor onset (500 ms) to the end of the trial (3000 ms). The odor was presented from 500 ms to 1500 ms. We calculated the time series for 36 E/I ratios. Note, that the decrease of the correlation coefficient during the odor duration is significant but it is masked by limited dynamical range of the graphs that also shows low correlations after odor offset.

The correlation coefficient (see analysis in Figure 4) and the Euclidean distance between PN activity vectors offer two distinct measures of the separation between odor representations. The correlation coefficient, which is the cosine of the angle between the 300–dimensional PN activity vectors representing the odors during each time window, is based on the normalized vectors and does not change in response to changes in the amplitude (the norm) of the vector. The correlation coefficient, therefore, depends mainly on the relative changes of the firing rates of individual PNs. Complex and odor-specific spatiotemporal PN dynamics would lead to rapid decreases in the correlation between odor responses. The correlation coefficient, however, would not change if the firing rates of all PNs increased or decreased proportionally. The Euclidean distance, on the other hand, can change both as a function of the angle and the amplitude of the activity vectors. With these tools we could examine how a change in the strengths of lateral excitation and slow inhibition would modify the distance between representations of similar odors. We anticipated that increasing lateral excitation would recruit more PNs that in turn would generate activity vectors with larger amplitudes. The distance between odor representations would therefore increase with increasing lateral excitation. In order to measure the distance between representations of similar odors (the Gaussian inputs to the ALs corresponding to each odor were separated by 5 units) we first constructed a PSTH for each of 300 PNs using 50 ms time bins. For each of these time bins the odor was represented by a 300–dimensional vector of PN activity. We calculated the Euclidean distance between these vectors in each time bin. The distance averaged across all time bins over the duration of an odor presentation was a measure of the distance between odor representations for a given network configuration. We found that an increase in lateral excitation increased the distance between odor representations (Figure 5a). As expected, an increase of slow inhibition led to the opposite trend, decreasing the amplitude of the response and, therefore, decreasing the distance between odors. A constant ratio of excitation to inhibition would correspond to the diagonal on this graph.

To determine effect of the ratio of excitation to inhibition 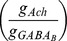 on the Euclidian distance and the correlation coefficient, we plotted the distance and correlation over time for different values of the excitation to inhibition ratio (Figure 5b). We found that changing the ratio of excitation to inhibition had a significant impact on the distance. Each row in the matrix (Figure 5b, middle panel) shows the Euclidean distance between odor response patterns for a value of the E/I ratio determined by the black trace (Figure 5b left panel). So, the top row corresponds to the minimal ratio (no excitation) and the bottom row illustrates distance for the maximal E/I ratio. Plotting all the time series in increasing order of the E/I ratio revealed that increasing the E/I ratio led to a very systematic increase in the distance.

To determine whether the correlation coefficient reflected a similar trend, for each value of lateral excitation and slow inhibition we calculated the correlation coefficient between 300–dimensional PN activity vectors generated as the network responded to the two similar odors independently. The correlation coefficients were determined during 50 ms epochs of time and the resulting time series were then plotted in increasing order of the E/I ratio (Figure 5b, right). We did not find any systematic changes in the rate of change of the correlation coefficient as a function of increasing E/I ratio. This suggests that changing E/I ratio leads to a systematic change in the mean amplitude of the PN responses (e.g., increase of the firing rates of all PNs); however, not necessarily to a systematic change in the relative balance of the individual PN firing rates. The individual firing rates may increase or decrease depending on the specific values of excitation and inhibition.

### Maximizing reliability and separability of odor representations

**Figure 6:**
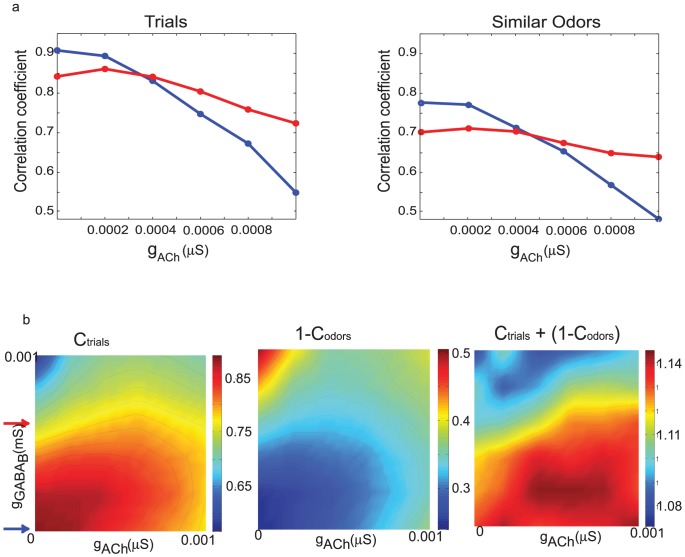
**a.** Top panels show the correlation coefficient as a function of lateral excitation for two different values of slow inhibition denoted by the colored arrows in the bottom panels. **b.** Image maps of the time averaged correlation coefficient as a function of lateral excitation and slow inhibition. The quantity to be maximized is calculated by summing the first two panels.

Animals are able to recognize an odor reliably each time it is presented despite the inevitable small variations in each presentation. Thus, our model of the olfactory system should be robust enough to avoid classifying each encounter with a given odor as unique. Correlations between the activity of PNs generated by one odor and that of another odor provide a measure of how well their representations may be distinguished by follower neurons. The olfactory system should therefore maximize correlations between multiple presentations of an odor while simultaneously minimizing correlations between representations of different odors. We found this could be achieved in our model with a balance of lateral excitation and slow inhibition. In Figure 6a the panels show changes in the correlation coefficient as a function of lateral excitation for two values of slow inhibition (red and the blue arrows in the panels Figure 6b below.) Increasing lateral excitation decreased the correlation between similar odors (Figure 6a, right), effectively augmenting the ability of the system to distinguish between these odors. However, it also decreased the correlation between different trials of the same odor presented with noise (Figure 6a, left), thus potentially sacrificing the reliability of responses. Increasing the amount of slow inhibition had different effects depending on the value of lateral excitation, further decreasing the correlation coefficient when lateral excitation was minimal but increasing the correlation coefficient when lateral excitation was stronger (compare relative position of red and blue curves in Figure 6a). A similar trend emerged from a comparison of correlations between multiple presentations of the same odor, indicating that balanced amounts of lateral excitation and slow inhibition are required to decorrelate different odors while maintaining the similarity of responses to different trials of the same odor.

We could readily achieve such a balance by maximizing the quantity (C_trials_+(1−C_odors_)) where C_trials_ is the correlation between multiple presentations of the same odor and C_odors_ is the correlation between the representations of different odors (see Methods for a detailed description of how these quantities were calculated). Even for small differences between odors (the peaks of the input to similar odors are shifted by only 5 units; the maximum possible shift between two odors is 150 units), C_trials_ and C_odors_ differed in magnitude. These differences implied that the term (C_trials_+(1−C_odors_)) was not uniform across the parameter space queried. Increasing the strength of both excitatory and slow inhibitory AL connections decreased correlations between trials (C_trials_; Figure 6b, left); however, at first, it led to an even faster decrease of correlation between similar odors, C_odors_. The latter corresponded to a rapid increase in the “anti-correlation” parameter (1**-** C_odors_; Figure 6b, middle), so the term (C_trials_+(1−C_odors_)) increased (Figure 6b, right). When values of excitation and inhibition were larger, the opposite trend emerged – the correlation between trials decreased faster than the correlation between odors (i.e., C_trials_ decreased faster than (1−C_odors_) increased), so the term (C_trials_+(1−C_odors_)) decreased (Figure 6b, right). Furthermore, when slow inhibition was increased while the lateral excitation was minimal, we observed an immediate decrease in correlations between multiple trials of the same odor (Figure 6b, left). However, an increase in slow inhibition in the presence of stronger lateral excitation (e.g., within the range 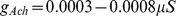, Figure, 6b, left) at first led to a small but reliable increase in correlations between trials of the same odor (note that the maximum of C_trials_ is found for positive values of 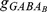 when 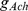 is positive); only when slow inhibition was further increased did the correlations decay. Together, these results illustrate an optimal range of excitation and inhibition in which gains in reliability across trials could be balanced against gains in the separation of similar odors (Figure 6b right). While the exact position of this maximum will vary with the choice of optimization function, these results indicate that both non-zero lateral excitation and inhibition are required to achieve a balance between the system's performance in the discrimination of similar odors and the reliable identification of the same odor across multiple trials with noise.

### Odor classification

**Figure 7:**
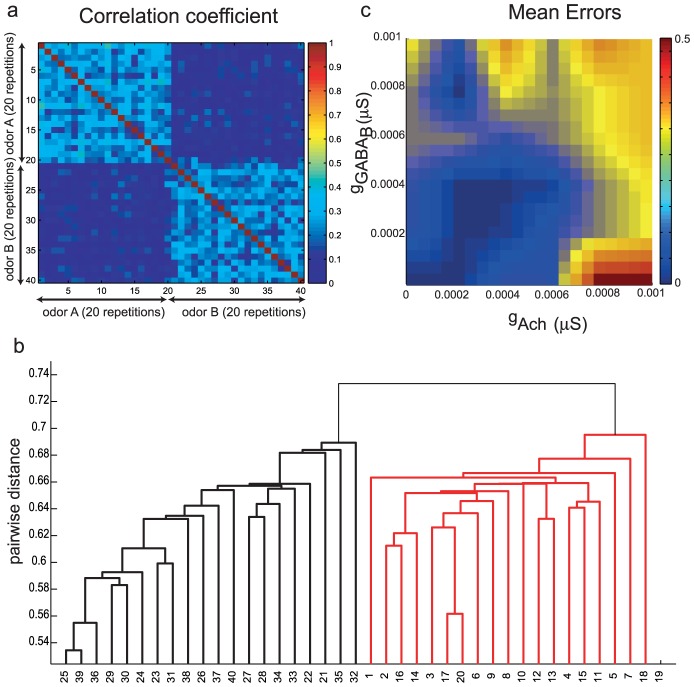
**a.** Average correlation coefficient between two odors. Each odor was repeatedly presented 20 times. The diagonal blocks show the correlation between trials of the same odor. The off–diagonal blocks with lower correlation coefficients provide a measure of the similarity between trials associated with different odors. **b.** Classification of responses by a hierarchical clustering algorithm. The bottom leaves of the tree represent each trial (40 trials across 2 odors). **c.** Mean error in classification of two similar odors.

The primary reason to compare the correlation between multiple presentations of the same odor versus correlations between similar odors was to understand the network mechanisms that enhance odor classification. In this section we examine how well a simple classification algorithm could differentiate similar odors despite realistic, noisy variations between multiple presentations of the same odor. The correlation between representations of the odor provides a useful metric of distance between representations. Figure 7a shows the average (over time) correlation between the responses of two similar odors (the inputs were separated by five units). Each odor was presented 20 times. The resulting correlation matrix consists of two diagonal blocks with higher correlations. These correspond to the correlation between different trials of the same odor. The off–diagonal blocks show a lower mean correlation coefficient between different odors. We used this correlation matrix as a measure of the pair–wise distance between individual presentations of odors. Since lower correlation indicates greater distance, we defined the pair–wise distance, 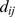, between representations 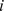 and 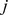 as 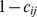, where 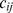 are elements of the correlation matrix. This distance was then used to cluster the representations into two groups using a hierarchical clustering algorithm (see Figure 7b and the Methods section). Since we knew *a priori* which representations corresponded to a particular odor, we could track the individual representations that were misclassified. We conducted the same analysis with nine more pairs of similar odors and different values of the relevant parameters, lateral excitation and slow inhibition. The average proportion of errors for these nine odor pairs is shown as a function of 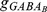 and 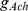 (Figure 7c). Optimal odor classification implies that the errors in classification were minimized. The pattern of errors that resulted from this classification could be broadly inferred from the measure C_trials_+(1−C_odors_) used in the previous section (see Figure 6b, right panel). Regions of minimum error in Figure 7c (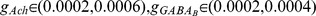) approximately coincided with the regions where C_trials_+(1−C_odors_) were high (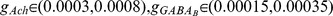), while the errors were maximal when C_trials_+(1−C_odors_) was low. Consistent with the correlation analysis (Figure 6b), when lateral excitation was present (e.g, for 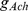
>0.0002 µS), the lowest classification error was obtained for non-zero values of slow inhibitory conductance (Figure 7c). A high error rate was obtained when lateral excitation was maximized and 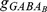 was set to zero (see bottom right corner in Figure 7c). For these parameter values, most PNs were recruited and overlaps between representations of the same odor and also between different odors were large. The exact value of 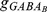 and 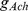 when the errors were minimized did not coincide exactly with the maximum of C_trials_+(1−C_odors_). However, a qualitative demarcation between regions of high and low error rates could be inferred from C_trials_+(1−C_odors_).

## Discussion

In insects, tens of thousands of ORNs converge onto a few hundred excitatory PNs and local inhibitory neurons in the AL [7]. Interactions among AL neurons contribute to the generation of spatiotemporal activity patterns that unfold over multiple timescales. This process may contribute to a progressive decrease in the overlap between representations of similar odors, a phenomenon that was originally described in the olfactory bulb of zebrafish [14]. In this study, using a realistic model of the locust AL, we examined the potential contributions of lateral excitatory and inhibitory connections to this temporal decorrelation.

Excitatory interneurons (eLNs) have recently been described in the *Drosophila* AL [17], [18], [21] and are likely to exist in locust as well, although no direct tests of their existence have yet been reported. In the *Drosophila* AL, a recently identified class of local cholinergic cells exhibits a widespread pattern of innervation that is not glomerulus–specific [19]. Electrophysiological recordings indicate that these cells tend to recruit PNs that receive zero or sub–threshold input from ORNs [18], potentially boosting the transmission of signals generated in the AL to follower neurons in the mushroom body [17]. In contrast to lateral excitation, slow inhibition [24] decreases the average activity of the AL over time scales spanning hundreds of milliseconds. (This gain modulation is distinct from the role of fast inhibition that, in concert with reciprocal excitation from PNs, is known to produce a fast oscillatory rhythm and synchronization of PN spikes over relatively fast time scales [15], [16], [24].)

We tested the hypothesis that both lateral excitatory and slow inhibitory connections, in proper balance, are required to achieve two apparently opposing goals during the processing the olfactory stimuli: to separate different but chemically similar odors (sensitivity, capacity) and to identify repeated instances of the same odor in a noisy environment (reliability).

We found that lateral excitation improves the sensitivity of the olfactory system by recruiting additional PNs that do not receive direct input from ORNs, thereby amplifying differences between the representations of similar odors [17], [18], [19], [25]. Increased sensitivity, however, could compromise the robustness of the AL's responses to multiple presentations of the same odor when noisy variations were included in the input. Slow inhibition could curtail the spread of PN activity and introduce reliable variations in spatiotemporal patterning over a time scale of hundreds of milliseconds. This effect depended on the level of lateral excitation. We found that increasing slow inhibition could lead to an increase in the correlation between trials of the same odor (increase in reliability) only when non-zero lateral excitation was implemented. Our study shows that both slow inhibitory connections and lateral excitatory connections mediated by local interneurons are required to enhance the decorrelation of similar odors while keeping the representations of odors robust across multiple encounters in the presence of noise. The decorrelation achieved by excitation and inhibition, in turn, enhances the ability of the olfactory system to classify odors; the error rate of classification was minimal in the presence of the balanced slow inhibitory and lateral excitatory connections.

ORNs are preferentially sensitive to some odors. This preference is manifest in the non-uniform firing rate distribution of ORNs with a high peak at low frequencies and a long tail over high frequencies [17]. The optimal distribution of neuron firing rates for odor discrimination would be one without peaks [26], [27], [28]. Such a response distribution may be achieved by a nonlinear transformation function, implemented in the AL, with a high gain for low firing rates that saturates for high firing rates, thereby employing the dynamic range of the PNs more effectively [17]. Our study suggests that this transformation may be achieved by the coordinated efforts of lateral excitation and slow inhibition in the AL. Indeed, in our model, increasing the strength of lateral excitatory connections mediated by local excitatory interneurons increased the fraction of PNs responding to an odor and also increased firing rates in many responding PNs. However, we found this effect could be balanced by slow inhibitory connections that reduced the firing rates of the most active PNs while maintaining a broad response profile across the PN population (Figure 2c). These results lead us to predict a strong link between odor decorrelation and the optimization of odor representations: maximal decorrelation is achieved in the AL network when firing rates of PNs are optimally distributed.

In our simulations we focused on the role network interactions play in decorrelating odor representations. Another contributor to the temporal patterning in the AL driving decorrelation appears to be the response dynamics of olfactory receptor neurons. Recent studies have characterized the temporal responses of ORNs by their response latency, rise time and adaptation to a prolonged odor presentation. Variations in these temporal properties, while not causing decorrelation in the responses of the ORNs themselves [29], may enable lateral inhibition in the AL to have this effect. Our model does not test the roles specific forms of lateral excitation and inhibition may play in the network, but rather argues more fundamentally that a balance of excitatory and inhibitory drive to PNs is required to enhance the ability of the system to decorrelate odor representations.

Our study suggests local excitatory and inhibitory interneurons of the insect AL provide balanced, functional circuitry that significantly reformats and optimizes odor representations in the AL network. While the effects of excitation and inhibition would cancel each other if averaged across the entire population of AL neurons, heterogeneous interconnectivity among the lobe's neurons would allow a given receptor to trigger responses dominated by inhibition in some PNs and by excitation in others. The combined effect of excitation and inhibition may provide an improved representation of the identity of an odor by being both robust against noise and sensitive to relatively small variations in the identities of active ORNs.

## Methods

The model network simulations were based on a realistic and robust model of the insect AL [15], [16] that had previously been used to establish the roles of fast and slow inhibition in the evolving spatiotemporal dynamics of the locust olfactory network. Our results show these features, including collective oscillatory dynamics and slow patterning, persist even when the ratio of excitatory and inhibitory neurons and the specific network architecture are varied.

### Neuron model

Individual projection and local inhibitory interneurons were modeled by a single compartment that included voltage and Ca^2+^ dependent currents described by Hodgkin–Huxley kinetics. Consistent with locust physiology, isolated PNs displayed overshooting Na^+^ spikes at a fixed frequency throughout DC stimulation, and local inhibitory neurons, by contrast, fired low amplitude Ca^2+^ spikes and displayed spike frequency adaptation caused by Ca^2+^–dependent potassium currents. A separate population of excitatory local interneurons with properties identical to the PNs was also simulated. The model AL network consisted of 300 PNs, 100 local inhibitory interneurons (LNs) and 50 local excitatory interneurons (eLNs) (Figure 1a).The ratio (3∶1) of the PNs to inhibitory LNs used in our model is based on known features of locust olfactory anatomy, which includes 830 cholinergic excitatory PNs and 300 inhibitory LNs [30]. Excitatory LNs have not been described (or comprehensively searched for) in locusts, but a number of indirect lines of evidence suggest some do exist. We included 50 eLNs in most of the simulations, but, by varying their numbers in a few control experiments, we found that the absolute number of eLNs did not significantly affect our results as long as we also compensated the strength of excitatory connections to ensure the same overall level of excitation per cell.

### Network interactions

Fast GABAergic (LN–PN, LN–eLN, and LN–LN connections) and nicotinic cholinergic synaptic currents (PN–LN, PN–eLN, eLN–LN) were modeled by first order activation schemes. Connection probabilities were as follows. P(PN–eLN) = 0.5, P(eLN–PN) = 0.1, P(PN–LN) = 0.5, P(LN–LN) = 0.5, P(LN–PN) = 0.5, P(eLN–LN) = 0.5, P(LN–eLN) = 0.5. These probabilities are constrained by estimates made from locust AL circuits [20], [30], [31]. Each locust LN receives excitatory input from 50–75% of the PNs as well as fast GABA_A_ type and slow GABA_B_ type inhibitory lateral inputs from 25–50% of the remaining LNs [30]. No self-inhibition has been reported in the locust AL. Each PN receives fast GABA_A_type and slow GABA_B_type inhibitory lateral inputs from 75% of the LNs (G.Laurent, personal communication). Probabilities of eLNs connections are presently unknown, however, varying their connection probability in our model produced effects similar to varying the number of eLNs (see above).

The AL network was simulated for a range of values of lateral excitation and slow inhibition. The maximal conductance denoting the total lateral excitation received by a given cell was set to a value ranging from 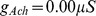 to 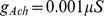 in steps of 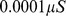. Similarly, the maximal conductance due to inhibitory GABA_B_ type receptors was set to values ranging from 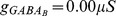 to 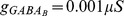 in steps of 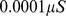.

### Input

The distribution of intensities provided to the PNs followed a Gaussian profile (Figure 1b). The standard deviation of the distribution was fixed at
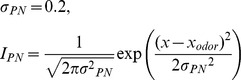
where 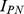 is the input to PNs. The variable x ranged from −1 to 1. The index of PN is related to x as follows, 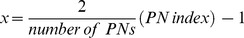. The identity of the odor is determined by the peak of the Gaussian input and is given by 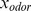. The input 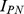 was truncated to zero for values of the Gaussian below 0.1 and scaled by a current of amplitude 0.00001 *nA*. The truncated Gaussian profile of the odor is shown in Figure 1b.

The time course of the stimulus was modeled as a current pulse with a rise time constant of 100 ms and a decay time constant of 200 ms. This was scaled by the factor 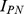 for each neuron. The form of the input that each neuron received is given below,
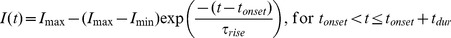


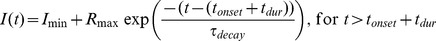
where, 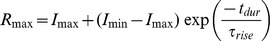. 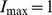 is the maximum amplitude of the input and 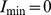 is the minimum. 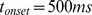 indicates the start of the stimulus with a rise time of 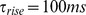 and a decay time constant of 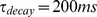. The stimulus decay began after 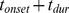 *ms*. In addition to the stimulus pulse, we also added a low amplitude noise term (∼5–10% of the stimulus amplitude). A similar input was also provided to the LNs and eLNs. This input was scaled by the term I_PN_ and was used to drive individual PNs.

Different odors were generated by progressively shifting the Gaussian input profile by5 unit steps. Similar odors were defined as odors with input profiles shifted by 5 units; dissimilar odors were shifted by 40 units (Figure 1b). For each pair (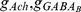) we stimulated the network with a sequence of 21 odors, each presented 10 times. Each presentation, termed a trial, lasted 1000 ms and consisted of an initial onset at 500 ms followed by a fast rise and a more gradual decay beginning at 1500 ms.

### Analysis

To calculate all measures of correlation we first generated a PSTH for individual neurons by determining the number of spikes produced by each neuron in consecutive 50 ms time bins that overlapped over 25 ms durations. The activity of the population of PNs (*n = 300*) during each time interval could then be characterized as a 300–dimensional vector. Each 3000 ms (500 ms before onset +1000 ms stimulus +1500 ms from offset) odor trial could then be represented by a 300×120 matrix with elements providing the number of spikes generated by the *i^th^* neuron during the *j^th^* time–bin for specified trial, odor and (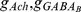) values. For a specified value of 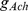 and 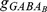 we first calculated the correlation between all pairs of odors (*n = 21*) to generate a 21×21 matrix for each time point. Each element of this matrix denoted the correlation between a 300–dimensional PN vector corresponding to the *i^th^* odor and the PN vector corresponding to the *j^th^* odor during a specific 50 ms time window. A similar set of matrices was also constructed to determine the correlation between the inputs to PNs ([14] employed similar measures to analyze the response of mitral cells in the zebrafish olfactory bulb). Next, we picked all pairs of odors separated by 5 units (similar odors are defined by *|i-j| = 5*, where *i* and *j* are the matrix indices). The value of the correlation coefficient was averaged across all such pairs of similar odors for different values of 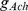 and 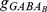. We also calculated the correlation between dissimilar odors (odor pairs with *|i-j| = 40*).

We then evaluated the correlation between multiple trials (*n = 10*) of the same odor. For each set of (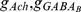) values we generated a sequence of 10×10 matrices denoting the correlation between PN vectors corresponding to different trials of the same odor. We then averaged the value of the correlation coefficient across all trials.

For each (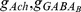) pair we determined the mean correlation coefficient between similar odors by averaging the correlation coefficients over the duration of the odor presentation. This provided us with a matrix of values C_odors_. Similarly, we calculated a matrix for the mean correlation between multiple trials of the same odor C_trials_. We then obtained the optimal value of 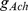 and 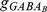 to minimize the correlation between similar odors while maximizing the correlation between multiple trials of the same odor by finding the location of the maximum value of the matrix, C_trials_+(1−C_odors_). The use of correlation coefficient over the Euclidean distance is preferred for this analysis as the correlation coefficient is already normalized between −1 and 1.

We performed the clustering analysis using the Matlab Statistics Toolbox. To cluster odor representations we first defined a distance between the spatiotemporal patterns generated in response to two odor stimulations. The two responses (*i* and *j*) could be the outcome of either different odors or different trials of the same odor. The distance between *i* and *j* was given by *d_ij_ = 1−c_ij_*, where *c_ij_* is the mean (averaged over the duration of the odor stimulus) correlation coefficient between the two response *i* and *j*. The greater the correlation (*c_ij_*), the smaller the distance between representations (*d_ij_*). Once the distance between every pair was calculated we sought to cluster the responses into two groups for each value of lateral excitation and slow inhibition. Since we know the identity of the odor that generated a particular response we could easily determine whether the response was classified accurately. The hierarchical classification algorithm (the ‘linkage’ function in Matlab) links pairs of proximate objects (based on the correlation distance defined above) into binary clusters that are then grouped together recursively to generate larger clusters until a hierarchical tree can be constructed. We then divided the odor representations into two classes by cutting the hierarchical tree at a level such that exactly two clusters were generated. Each of these clusters (say cluster 1 and cluster 2) was then assigned to one of the odors (say odor A or odor B respectively). We then determined the number of odor representations that were misclassified (*N_err1_*). We then switched the assignment (cluster 2 was assigned to odor A and cluster 1 was assigned to odor B). The number of errors (*N_err2_*) was computed again. The actual number of errors was then defined as *N_err_ = min(N_err1_, N_err2_)*. The error proportion *N_err_/N_total_*, where *N_total_* is the total number of odor representations is shown in Figure 7. The chance level is 0.5.
